# Analysis of Differences in Phenology Extracted from the Enhanced Vegetation Index and the Leaf Area Index

**DOI:** 10.3390/s17091982

**Published:** 2017-08-30

**Authors:** Cong Wang, Jing Li, Qinhuo Liu, Bo Zhong, Shanlong Wu, Chuanfu Xia

**Affiliations:** 1 State Key Laboratory of Remote Sensing Science, Institute of Remote Sensing and Digital Earth, Chinese Academy of Sciences, Beijing 100101, China; wangcong418@126.com (C.W.); zhongbo@radi.ac.cn (B.Z.); wsl0579@163.com (S.W.); xiachuanfu2897@163.com (C.X.); 2 University of Chinese Academy of Sciences, Beijing 100049, China

**Keywords:** remote-sensing phenology product, MODIS-EVI, GLASS-LAI, ground observations, comparison

## Abstract

Remote-sensing phenology detection can compensate for deficiencies in field observations and has the advantage of capturing the continuous expression of phenology on a large scale. However, there is some variability in the results of remote-sensing phenology detection derived from different vegetation parameters in satellite time-series data. Since the enhanced vegetation index (EVI) and the leaf area index (LAI) are the most widely used vegetation parameters for remote-sensing phenology extraction, this paper aims to assess the differences in phenological information extracted from EVI and LAI time series and to explore whether either index performs well for all vegetation types on a large scale. To this end, a GLASS (Global Land Surface Satellite Product)-LAI-based phenology product (GLP) was generated using the same algorithm as the MODIS (Moderate Resolution Imaging Spectroradiometer)-EVI phenology product (MLCD) over China from 2001 to 2012. The two phenology products were compared in China for different vegetation types and evaluated using ground observations. The results show that the ratio of missing data is 8.3% for the GLP, which is less than the 22.8% for the MLCD. The differences between the GLP and the MLCD become stronger as the latitude decreases, which also vary among different vegetation types. The start of the growing season (SOS) of the GLP is earlier than that of the MLCD in most vegetation types, and the end of the growing season (EOS) of the GLP is generally later than that of the MLCD. Based on ground observations, it can be suggested that the GLP performs better than the MLCD in evergreen needleleaved forests and croplands, while the MLCD performs better than the GLP in shrublands and grasslands.

## 1. Introduction

Phenology is the study of the timing of recurring biological events, the causes of their timing with regard to biotic and abiotic forces, and the interactions among phases of the same or different species [1]. Sprouting and flowering of plants in spring, color changes of leaves in fall, bird migration and nesting, insect hatching, and animal hibernation are all examples of phenological events [2]. Phenology is an important indicator of global change and the carbon cycle because of its direct effects on vegetation photosynthesis, carbon sequestration and land–atmosphere water and energy exchange [3]. Vegetation phenology indicates the responses of plants to seasonal and interannual variations of climate, hydrology, soil and anthropogenic factors [4]. Vegetation phenology also affects the climate system by influencing the seasonality of albedo; surface roughness length; canopy conductance; and fluxes of water, energy, CO_2_ and biogenic volatile organic compounds [5]. The extraction of vegetation phenology is important for research on global climate change and terrestrial ecosystems [6]. The phenology products retrieved from remote-sensing data have been widely applied in many fields, such as climatic change [7,8,9,10], biomass monitoring [11,12,13,14] and farm management [15,16,17,18].

Remote-sensing vegetation phenological metrics are generally derived from satellite time-series data of vegetation parameters. Vegetation indexes (VIs) are one of the most commonly used parameters, including the normalized difference vegetation index (NDVI) [4,19,20,21] and enhanced vegetation index (EVI) [22,23,24]. Generally, the NDVI tends to lose sensitivity over dense canopies because of saturation [25], while the EVI has a larger dynamic range than the NDVI and is more resistant to atmospheric and soil background effects [26]. Many studies have explored other VIs to indicate the growing season transitions, such as the soil adjusted vegetation index (SAVI) [27], MERIS terrestrial chlorophyll index (MTCI) [28], perpendicular vegetation index (PVI) [9] and wide dynamic range vegetation index (WDRVI) [25]. While VI is an integrated indicator of the vegetation, the soil background, and the observation-illumination geometry, some vegetation physiological parameters have also been used to detect vegetation phenology, such as the leaf area index (LAI) [3], the fraction of photosynthetically active radiation (fPAR) [29] and albedo [30]. Compared with the VIs, these parameters provide more explicit information on the biophysical characteristics of vegetation changes. Among these parameters, the LAI is most widely used because of the availability of multiple remote-sensing products and its direct indication of physical and biological processes related to vegetation dynamics at global and regional scales, such as energy exchange and water and carbon cycling [31,32].

In addition, many methods have been developed to monitor vegetation phenology using satellite time-series data. The methods can be grouped into six classes: threshold-based, delayed moving average, curve fitting, curve derivative, phenological cumulative frequency and principal component analysis. The threshold-based method defines the start of season (SOS) using a predefined or relative reference value [4,33,34]. The delayed moving-average method determines the SOS based on an autoregressive moving average (CARMA) model [35]. The curve-fitting method fits the time-series remote-sensing data with a smooth model and then determines vegetation phenology using the fitted function [22,36,37,38,39,40,41]. The curve-derivative method defines the time point of the time-series curve with the maximal second derivative value as the SOS [42]. The phenological cumulative-frequency method uses field phenology observations and the phenological frequency distribution pattern to determine the threshold of plant phenology events [43]. The principal component analysis method extracts vegetation phenology based on the characteristics of the first principal component of the datasets employing empirical orthogonal function (EOF) decomposition with NDVI [44].

Compared with the strong demand for vegetation phenology mapping for global and regional research and applications, the development of remote-sensing vegetation phenology products has been slower than other remote-sensing parameters, such as the LAI and VIs. Few regional-to-global remote-sensing vegetation phenology products have been generated despite the value of vegetation phenology for Earth-system monitoring and modeling [45,46]. The MODIS (Moderate Resolution Imaging Spectroradiometer) Global Land Cover Dynamics product (MLCD) is the only standard global product and is generated by NASA from MODIS-EVI time-series data [22,47].

The MLCD is based on the piecewise logistic model, which is the most widely used model in remote-sensing phenology extraction as it can effectively restrain the noise effect and requires no threshold or empirical constraints. Scholars have performed some validations for the MLCD. Comparisons with ground data suggest that the MLCD performs well, but the end-of-season metrics associated with vegetation senescence and dormancy have higher uncertainties than the start-of-season metrics [47]. In evergreen forests, the MLCD is unable to provide consistent phenologic patterns, and considerable data are missing, especially in tropical rainforests [48]. Against this background, Verger et al. [49] characterized the baseline phenology of the vegetation at the global scale from the GEOCLIM climatology of the LAI estimated from the 1-km SPOT-VEGETATION time series, where the start- and end-of-season were identified using 30% and 40% thresholds of the LAI amplitude values, respectively. Although the spatial patterns of the LAI-derived phenology agreed well with those from the MODIS-EVI and -NDVI, the timing of the start, end, and length of season differed by approximately one month at the global scale. The higher uncertainties appear in areas with limited seasonality expressed in the satellite signal and systematic biases due to the differences in the methodologies and datasets. Some other studies have also shown significant discrepancies in vegetation phenological metrics derived from time series of different vegetation parameters [50,51,52]. However, it is not clear which data sources and vegetation parameters are best suited for extracting vegetation phenological metrics accurately.

While EVI and LAI are the most widely used vegetation parameters that can be used for remote-sensing phenological extraction, this paper aims to assess the differences in phenological information extracted using the EVI and LAI time series and to determine whether either EVI or LAI time series performs well for all vegetation types over a large scale. The objective is to compare the phenology detection results in China from the MODIS-EVI and the GLASS-LAI. To achieve this objective, the GLASS-LAI phenology product (GLP) was generated using the same algorithm as the MODIS-EVI phenology product (MLCD) in China from 2001 to 2012. The two phenology products were compared in different vegetation types in China and then evaluated using ground observations from forests, grasslands and croplands. Our work lays the foundation for uniting multisource data and for improving remote-sensing phenology products in the future.

## 2. Data and Methods

### 2.1. Phenology Extraction Based on the EVI and LAI

The MLCD product was used as the vegetation phenology extracted from the EVI for analysis in the paper. The MLCD provides yearly 500-m resolution global estimates of vegetation phenology [47] and is available for the public at https://lpdaac.usgs.gov/dataset_discovery/modis/modis_products_table/mcd12q2. The MLCD identifies phenophase transition dates based on the logistic functions fit-to-time series of the EVI, which were calculated from composite 8-day normalized BRDF (Bidirectional Reflectance Distribution Function)-adjusted reflectance data [26]. Specifically, a time series of the EVI is assembled for each pixel, the data undergo a gap-filling and smoothing process, periods of sustained EVI increase or decrease are identified, logistic models are fit to the time series, and transition dates are identified as local maxima and minima in the rate of curvature change of the fitted logistic function [22]. The MLCD has four layers corresponding to key phenological stages: onset greenness increase, onset greenness maximum, onset greenness decrease, and onset greenness minimum. MLCD images over China from 2001 to 2012 were acquired for the study. Due to its different spatial resolution from the GLP, the MLCD was upscaled to 1000 m by averaging the value from every four pixels for statistics of different values between GLP and MLCD. But in other analyses, MLCD was used with its original spatial resolution.

The vegetation phenology extracted from the LAI was generated from the GLASS-LAI (http://glass-product.bnu.edu.cn/) that provides yearly 1000-m resolution global estimates of vegetation phenology, which is called the GLASS-LAI-based phenology product (GLP). GLASS-LAI was estimated from MODIS and CYCLOPES LAI time series using general regression neural networks, with 8-day temporal resolution and 1-km spatial resolution. Based on multitemporal data, GLASS-LAI are spatially and temporally continuous with no gaps in time series and no high-frequency temporal variability. As a result of cloud, atmospheric and snow contamination, Savitzky–Golay filtering was used to smooth and gap-fill the CYCLOPES LAI. The MODIS reflectance was reprocessed to remove remaining effects of cloud contamination and other factors [53]. The GLP applied the same algorithm as MLCD (the piecewise logistic model) to fit the LAI time series, which is a flexible, repeatable, and realistic means of monitoring seasonal and interannual vegetation dynamics from remote-sensing data at large scales. The piecewise logistic equation is represented as Equation (1):

(1) $$y\left(t\right)=\frac{c}{1+e^{a+bt}}+d$$

where *t* is time in days, *y*(*t*) is the LAI value at time *t*, *a* and *b* are fitting parameters, *c* + *d* is the maximum LAI value, and *d* is the initial background LAI value. GLP products with 1-km resolution were generated over China from 2001 to 2012.

**Figure 1:**
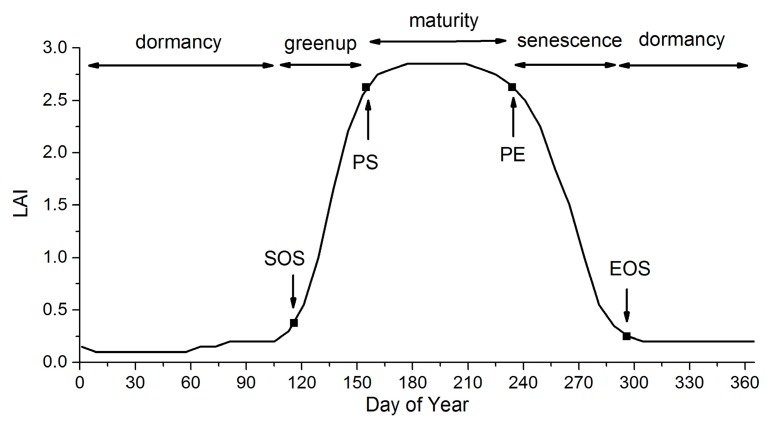
The phenological metrics of vegetation dynamics.

For both the MLCD and GLP products, the vegetation dynamics can be divided into four stages: greenup, maturity, senescence and dormancy [22], as shown in the Figure 1. The key transition dates define the main phases of vegetation dynamics, including the start of the growing season (SOS), the start of the peak (PS), the end of the peak (PE) and the end of the growing season (EOS): (1) SOS is the date of onset of photosynthetic activity; (2) PS is the date when the greenness reaches its maximum; (3) PE represents the date when the photosynthetic activity and the greenness begin to decrease rapidly; and (4) EOS is the date when the physiological activity approaches zero [14,54].

### 2.2. Remote-Sensing Auxiliary Datasets

**Figure 2:**
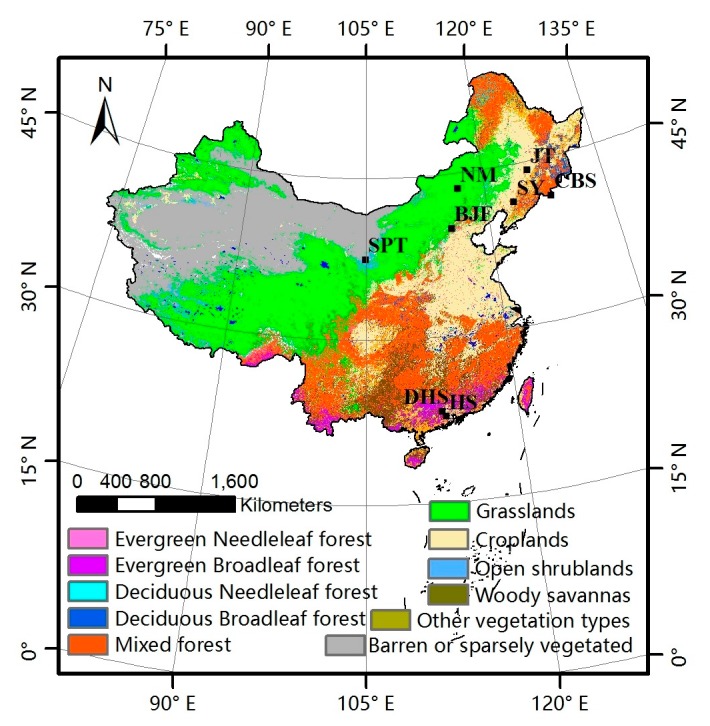
Land cover map and phenological observation sites distribution in China.

The nadir BRDF-adjusted reflectance (NBAR) data from MODIS (MCD43A4) were used to compute the EVI, as shown in Equation (2). The MODIS-EVI products and the GLASS-LAI products were used to compare the difference of vegetation growing states reflected by the LAI and EVI time series. Besides, the land cover type (MOD12Q1) was downloaded from NASA’s Earth Observing System Data Center website. The MOD12Q1 was used to identify the land cover type over pixels, as shown in Figure 2.

(2) $$\mathrm{EVI}=2.5\times \frac{\rho_{NIR}-\rho_{RED}}{\rho_{NIR}+6.0\rho_{RED}-7.5\rho_{BLUE}+1}$$

where $\rho_{NIR}$, $\rho_{RED}$ and $\rho_{BLUE}$ are the reflectances of near infrared, red and blue bands respectively.

### 2.3. Ground Phenological Observations

The ground phenological observations were taken as the true values to evaluate the accuracy of the phenology products. However, the ground-based phenology was observed in a number of individual plants, while the remote-sensing phenology represented the integrated phenological characteristics of a plant community in one pixel. Ground validation of remote-sensing measurements with coarse resolution entails considerable difficulties. To improve the reliability of the statistical analysis based on ground observations, ground phenological observations meeting the following requirements were selected from the China Meteorological Administration (CMA) and the Chinese Ecosystem Research Network (CERN):

(1) Spatial representation. The poor relationship between ground and satellite phenology due to data-scale issues is a drawback of satellite phenology because of the small chance of a single-point ground observation being representative of an entire area at the remote-sensing scale (typically ≥1 km in remote-sensing phenological studies) [4,35]. Consequently, the phenological homogeneity and subdued topography of field sites must be ensured in comparison with the remote-sensing data [41]. The phenological homogeneity requires the phenophases of dominant species at one site be similar (less than 30 days), and the topography is checked by Google Earth to avoid sites in mountains as far as possible. The dominant species were selected after considering the distribution and quantity of the community based on the instruction files from CERN and CMA.
(2) Data integrity. The selected ground sites should have phenological phase continuity and few missing records.

The ground phenological observations were selected according to the above requirements, as shown in Table 1. Each site corresponds to a typical vegetation type, including evergreen needleleaved forest (ENF), evergreen broadleaved forest (EBF), deciduous needleleaved forest (DNF), deciduous broadleaved forest (DBF), shrub, grass and crop. Rice was chosen as a representative crop with two sites, because the SY (Shenyang) site from CERN can provide harvest and JT (Jiutai) site from CMA can provide grain-filling. The mean phenological dates of the dominant species were calculated and treated representative of this region. The distribution of the ground phenological observation sites is shown in Figure 2.

### 2.4. Methods for Evaluating the Phenology Products

To analyze the differences in the GLP and the MLCD, the mean phenological metrics for each pixel in each year were calculated as the spatial pattern of China. Furthermore, the mean and standard deviation of the difference values between the two products were calculated based on land cover type data. In addition, the mean bias (MB) and mean absolute bias (MAB) were used to evaluate the remote-sensing phenological metrics based on ground-station observations [55], as shown in Equations (3) and (4).

(3) $$MB=\frac{\sum_{i=1}^{N}\left(rs-obs\right)}{N}$$

(4) $$MAB=\frac{\sum_{i=1}^{N}\left|rs-obs\right|}{N}$$

where *rs* is the remote-sensing phenological date; *obs* is the ground observation; and *N* is the number of *rs* or *obs*.

## 3. Analysis and Results

### 3.1. Comparison between the GLP and the MLCD over China

**Figure 3:**
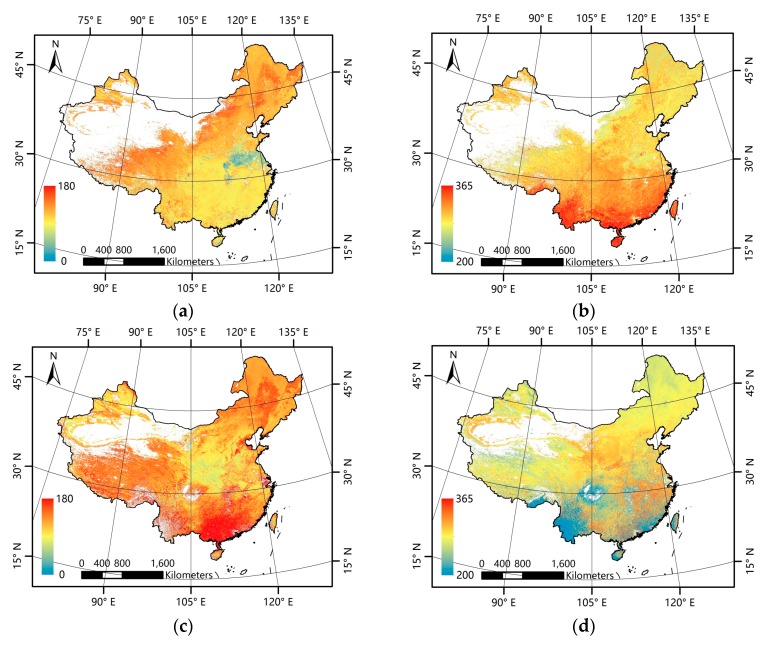
Patterns of mean phenological metrics of the GLP and the MLCD. (**a**) SOS of GLP; (**b**) EOS of GLP; (**c**) SOS of MLCD; (**d**) EOS of MLCD.

Figure 3a–d presents the mean phenological metrics for China for the 2001–2012 period derived from the GLP and MLCD data. In general, the GLP and the MLCD have consistent patterns in the north of China, but inconsistent patterns are exhibited in the south. For both the GLP and the MLCD, the missing data exist inevitably and vary in different regions.

#### 3.1.1. Missing Data

**Figure 4:**
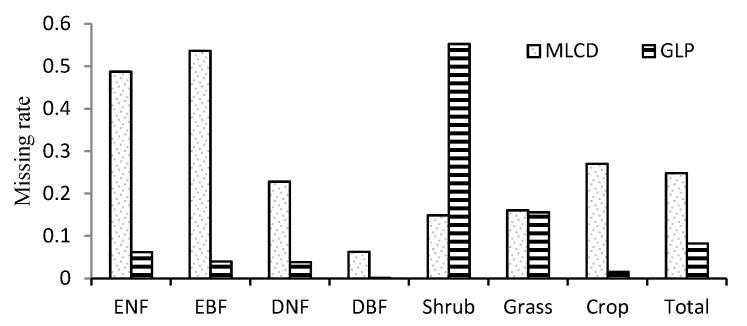
Missing ratios in different vegetation types.

In general, the ratio of missing data is 8.3% for the GLP, which is obviously less than the 22.8% for the MLCD, as shown in Figure 4. The missing ratios of the MLCD and the GLP differ obviously over different vegetation types. For the MLCD product, the missing ratio is highest in EBF (53.6%), followed by ENF (48.73%), which is much higher than that of GLP (6.2% and 4.0% respectively). Nevertheless, the missing ratio of shrub is the highest for GLP (55.3%), which is higher than that of MLCD (14.9%). In addition, the missing ratio of MLCD in crop is relatively high, especially for the double-cycle crop in the North China Plain (Figure 2); invalid results are also treated as missing data.

**Figure 5:**
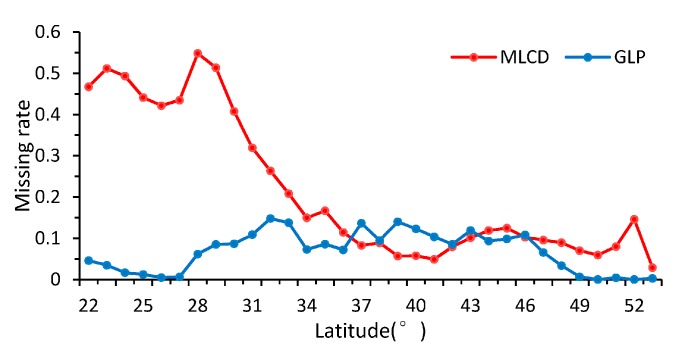
Missing ratios in different latitudinal bands.

**Figure 6:**
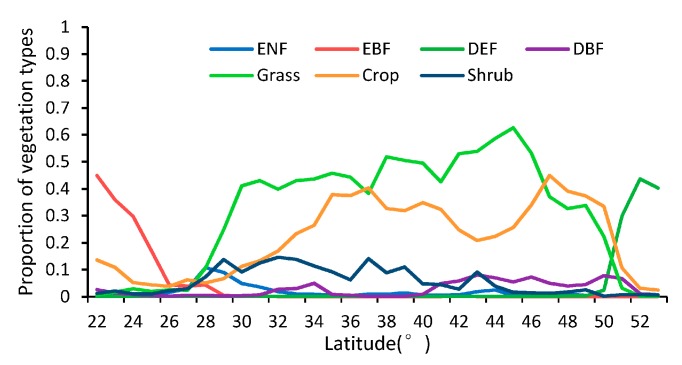
Proportion of vegetation in different latitudinal bands.

As shown in Figure 5, the patterns of the missing ratios depend strongly on the latitude between 22° N and 53° N in China. In general, the missing ratios of the MLCD are higher than those of the GLP along each latitude zone except from 37° N to 43° N. The missing ratio of the MLCD gradually decreases as the latitude decreases, especially in midlatitude zones (30° N–53° N), and remained inordinately high between 22° N and 30° N (approximately 50%), where much data are missing in the Sichuan Basin (as shown in Figure 2). However, the missing ratio of GLP largely depends on desert vegetation. For the GLP, the latitudinal distribution of missing data is consistent with the distribution of grass or shrub, as shown in Figure 5 and Figure 6.

There are several factors causing the missing data. The first is incomplete of time-series source data caused by clouds or snow. Cloud cover severely compromises the quality of NBAR EVI time series in extensive areas of tropics and subtropics [56], as a consequence, many phenological metrics are missing in these areas. In addition, the seasonal change characteristics of evergreen forest are not obvious, so it is hard to extract the phenology information of EBF and ENF. While GLASS-LAI exhibits spatial-temporal continuity [53], there is no gaps between GLASS-LAI time series. This is the main reason why the missing ratio of GLP is less than that of MLCD generally, but the missing ratio of GLP in desert vegetation (e.g., shrub) is relatively high because the range of the seasonal variation in the LAI time series is small. Therefore, fitting by the logistics method fails easily.

#### 3.1.2. Difference Comparison between the GLP and the MLCD

**Figure 7:**
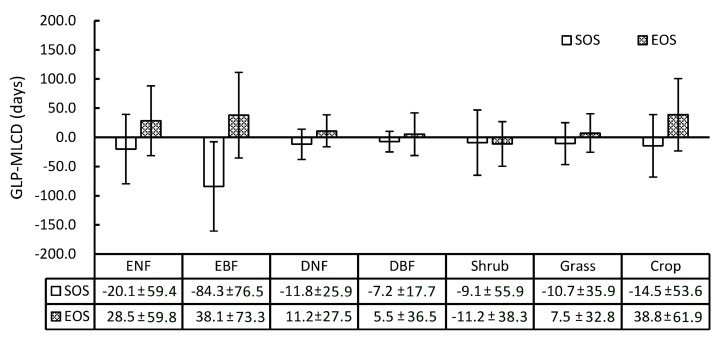
The means and standard deviations of the difference values between the GLP and the MLCD in different vegetation types.

Specifically, the difference between the GLP and the MLCD varies among different vegetation types, as shown in Figure 7. Overall, the SOS of the GLP is earlier than that retrieved by the MLCD in most vegetation types, while the EOS of the GLP is later, including shrub. Among the vegetation types, the difference between the GLP and the MLCD in deciduous broadleaved forest is the smallest, among which the difference in SOS is smaller than that of EOS (the means and standard deviations are −7.2 ± 17.7 and 5.5 ± 36.5, respectively). The greatest difference occurs in evergreen broadleaved forest, among which the difference in SOS is larger than that of EOS (the means and standard deviations are −84.3 ± 76.5 and 38.1 ± 73.3, respectively). In addition, the difference in SOS or EOS between the GLP and the MLCD in croplands is relatively large (the means and standard deviations are −14.5 ± 53.6 and 38.8 ± 61.9, respectively), as double-cycle crops are poorly detected in MLCD.

**Figure 8:**
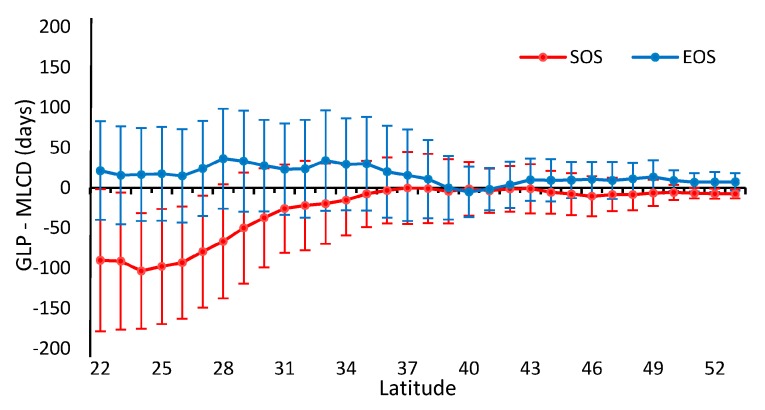
The difference values between the GLP and the MLCD in different latitudinal bands.

Obviously, the differences between the GLP and the MLCD become stronger as the latitude decreases, as shown in Figure 2 and Figure 8. The difference between the GLP and the MLCD largely depends on different vegetation types. The greatest difference between the GLP and the MLCD is found below 26° N, where EBF is mainly distributed. As the ratio of EBF decreases, the difference decreases. Because DBF is mainly distributed above 40° N, the difference between the GLP and the MLCD become small above 40° N. The difference in the SOS below 40° N is larger than that in the EOS, and the differences in the SOS and the EOS are similar above 40° N.

### 3.2. Accuracy Assessment Based on Field Data

**Figure 9:**
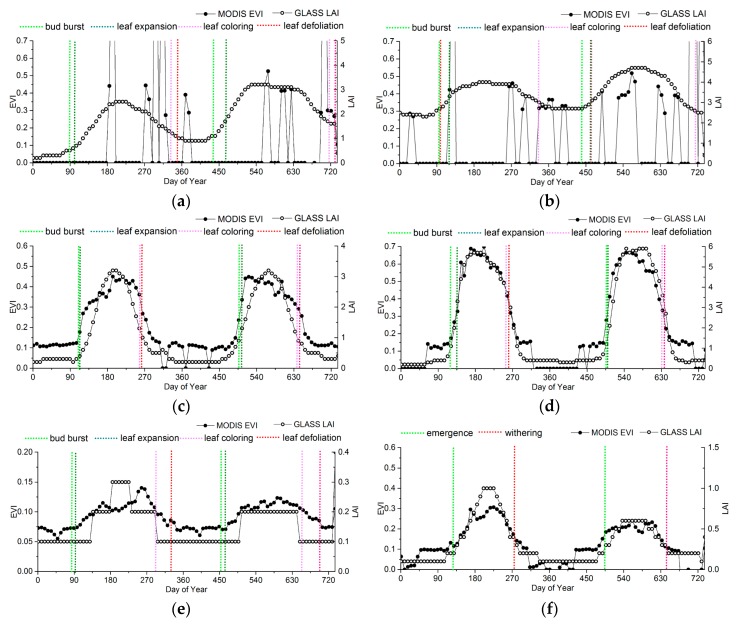

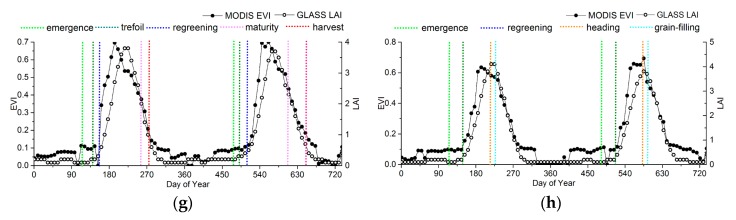
Comparison between the LAI and the EVI time series for different vegetation types based on the ground observations. (**a**) HS (ENF); (**b**) DHS (EBF); (**c**) BJF (DNF); (**d**) CBS (DBF); (**e**) SPT (shrub); (**f**) NM (grass); (**g**) SY (rice); (**h**) JT (rice).

To analyze the variances and relationships between ground observations and the phenological information extracted from the MODIS-EVI and GLASS-LAI products, the EVI and LAI time series at representative sites were chosen as reference to compare the phenological results, as shown in Figure 9. The information from ground phenology observation sites is presented in Table 1.

For evergreen forests, significant discrepancies occur in the LAI and EVI time series (Figure 9a,b). Obviously, irregular fluctuations exist in the EVI of ENF and EBF, which are more likely due to a pattern of noise caused by clouds in the EVI data structure rather than to vegetation growing changes. Thus, the phenological metrics extracted by MODIS-EVI may be discontinuous, or even missing, especially in the tropical climate zone. Apart from noise, in many areas with evergreen vegetation, the seasonal change characteristics in EVI is too subtle to retrieve phenology.

For deciduous forests, the LAI time series agree with the EVI time series overall, but the LAI time series have fewer fluctuations than the EVI time series in the peak (Figure 8c,d). The differences between LAI and EVI time series in DNF is larger than that in DBF. In DNF, although the LAI start to increase earlier than the EVI, the EVI is more sensitive than the LAI during the greenup. Therefore, the EVI generally reaches maturity faster than the LAI. And also, the EVI start to decrease later than the LAI, but decreases rapidly to the minimum.

The LAI and the EVI of shrublands are relatively lower. Although the range of seasonal variation of the EVI time series is smaller than that of the LAI, the precision of the current LAI products is 0.1, which is easier for presenting the “ladder” time series than the EVI product, whose precision is 0.01. Thus, the EVI shows the subtle dynamic changes of grasslands more clearly. As shown in Figure 9e, the inflection point of the EVI time series is close to bud burst and leaf defoliation of shrub. When using the LAI, it is difficult to show the subtle dynamic changes, causing a time offset in shrub phenology. Especially for shrubs in desert, the ranges of the seasonal variations in EVI or LAI are so small that the logistics model is difficult to fit to extract phenological information.

For croplands, although the LAI start to increase earlier than the EVI, the EVI is more sensitive to vegetation growth than the LAI during the greenup. Therefore, he EVI generally reaches maturity faster than the LAI, but the peak of the LAI time series is closer to the ground observations, as shown in Figure 9g,h.

In order to take the ground phenology observations as the truth values to evaluate the accuracy of the phenology products, the phenophases observed from ground observations corresponding to remote-sensing phenological metrics are determined, as shown in Table 2. All the observed ground phenology stages are the beginning of the phenophases in both CERN and CMA, so the phenophases observed from ground observations that were closest to the remote-sensing phenological metrics were chosen based on Figure 9. According to previous studies, the key remote-sensing phenological metrics might correspond to the four growing stages for crop [57,58]. For herbs and deciduous broadleaf trees, no phenophases correspond to PS and PE.

**Figure 10:**
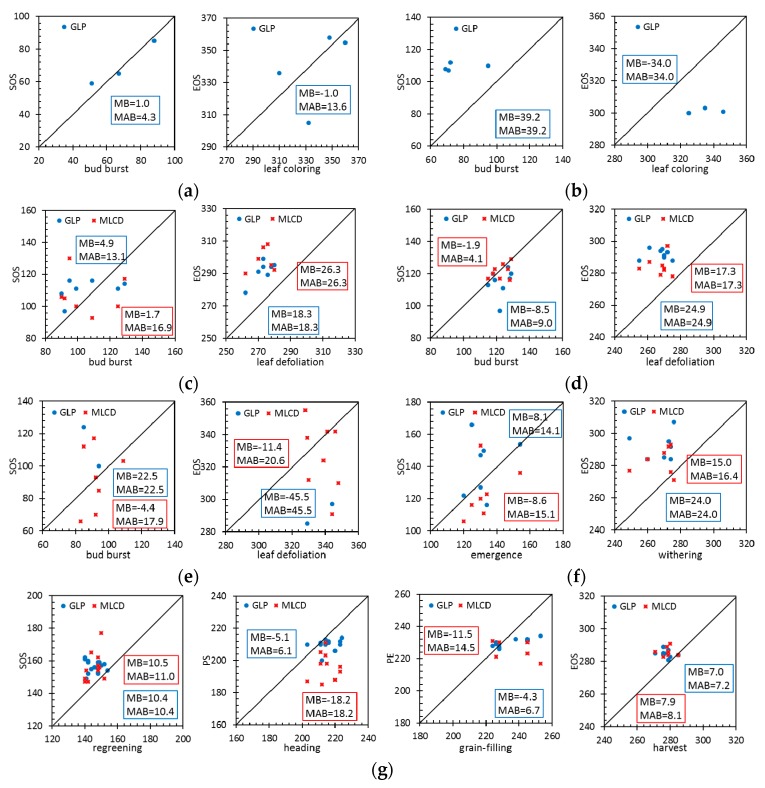
Comparison between the GLP and the MLCD for different vegetation types based on the ground observations. (**a**) HS (ENF); (**b**) DHS (EBF); (**c**) BJF (DNF); (**d**) CBS (DBF); (**e**) SPT (Shrub); (**f**) NM (Grass); (**g**) SY + JT (rice).

Figure 10 compares the MB and MAB values between the remote-sensing phenological metrics and the ground data at typical vegetation sites.

For evergreen forests, the phenological metrics of MLCD are missing at the HS and DHS sites; only the phenological metrics of GLP are presented, as shown in Figure 10a,b. Specifically, LAI can extract the phenology information of ENF especially for SOS with high accuracy. The MAB of the GLP for the SOS is 4.3 days (MB = 1.0 day), and the MAB of the GLP for the EOS is 13.6 days (MB = −1.0 day). However, GLP performs poorly in EBF, the MAB of the GLP for the SOS is 39.2 days (MB = 39.2 days), and the MAB of the GLP for the EOS is 34.0 days (MB = 34.0 days).

For deciduous forests, the accuracy of GLP or MLCD for SOS is higher than that for EOS, as shown in Figure 10c,d. The SOS of MLCD in DBF is very close to the 1:1 line, suggesting that the SOS that extracted by EVI is in good agreement with ground observations. Specifically, the MAB of the MLCD for the SOS is 4.1 days (MB = −1.9 days), and the MAB of the GLP for the SOS is 9.0 days (MB = −8.5 days). The EOS of GLP is later than that of MLCD, and their MABs are 22.9 days and 17.3 days respectively.

For shrublands, because LAI is hard to extract the phenology information in desert at SPT (Shapotou) site, only a few phenological metrics of GLP are presented, as shown in Figure 10e. Specifically, the MABs of the MLCD for the SOS and EOS are 17.9 days (MB = −4.4 days) and 20.6 days (MB = −11.4 days). Compared to shrublands, the MABs of the MLCD and the GLP are smaller. The MAB of the MLCD for the SOS is 15.1 days (MB = −8.6 days), and the MAB of the GLP for the SOS is 14.1 days (MB = 8.1 days). The EOS of GLP is later than that of MLCD, and their MABs are 24.0 days and 16.4 days respectively.

For croplands, the distributions of MLCD for SOS and EOS are similar to that of GLP. The MABs of the MLCD and the GLP for the SOS are 11.0 days (MB = 10.5 days) and 10.4 days (MB = 10.4 days) respectively. The MABs of the MLCD and the GLP for the EOS are 8.1 days (MB = 7.9 days) and 7.2 days (MB = 7.0 days) respectively. However, the accuracy of GLP for the PS is much higher than that of MLCD, since the MAB of the GLP and the MLCD for the PS is 6.1 days (MB = −5.1 days) and 18.2 days (MB = 18.2 days) respectively. Compared to the PS, the MABs of both phenology products are larger for PE.

## 4. Discussion and Conclusions

This paper assessed the differences in phenological information extracted from the EVI and LAI time series and explored whether the EVI or the LAI time series performs well for all vegetation types over a large scale in China. The GLP and the MLCD were compared in different regions and evaluated based on ground observations.

The missing ratio of the MLCD is more than that of the GLP overall. The main reason is incompletion of time-series source data caused by clouds or snow for the MLCD product. While GLP is not affected by clouds or snow, because the GLASS-LAI is provided with spatial-temporal continuity, there are no gaps in time series and no high-frequency temporal variability. However, the missing ratio of GLP in desert vegetation (e.g., shrub) is relatively high. Because the range of seasonal variation of LAI time series is small, fitting by the logistics method easily fails. Besides, in many areas with evergreen vegetation, the annual variation in EVI is too subtle to retrieve phenology.

Accuracy assessment is always an important concern in any remote-sensing-based analysis, especially one performed at a large scale. To improve the reliability of the statistical analysis based on ground observations, we selected sites based on the requirements of spatial representation and data integrity. The accuracy assessment based on representative sites in this paper show that both phenology products can provide the best accuracy with ground observations for the SOS of DBF and the EOS of rice. Because the leaf defoliation period for DBF lasts for one or two months, and the EOS extracted from LAI may be close to the end of leaf defoliation, a lag can occur between the EOS and the start of leaf defoliation from ground observations. Besides, previous research has shown that the end-of-season metrics of the MLCD associated with vegetation senescence and dormancy are highly uncertain [47,56]. In this paper, it is shown that the EOS in croplands has less uncertainties than that in forests. Thus, it can be inferred that the uncertainties of EOS in forest may come from its complex structure and composition, as the composition of cropland is relatively homogeneous. For grasslands or shrublands, the range of seasonal variation is relatively small, the logistics model couldn’t fit well with EVI or LAI time series; as a consequence, the accuracy of grasslands or shrublands is worse than that of deciduous forests or croplands. LAI is easier for presenting the “ladder” time series than the EVI and even failing in fitting when the maximum of LAI is less than 0.3. In addition, the growth of grass or shrub in arid regions or desert is sensitive to precipitation, which may result in irregular fluctuations in EVI time series. Our analysis also shows that the GLP performs better than the MLCD in ENF and croplands, especially for the PS and PE of crop. Although GLP has less missing data in EBF, the disagreement exist between vegetation phenological metrics and ground observations. However, the MLCD performs better than the GLP in shrublands and grasslands. The precision of the current LAI product is 0.1, indicating that it does not show subtle dynamic changes as well as the EVI product, whose precision is 0.01.

It is noted that MLCD is derived from 500-m, 8-day composite EVI data; the GLP is derived from 1000-m, 8-day composite LAI data; while ground observations are daily point-based observations. The temporal and spatial scales of ground observations make them difficult to compare directly with remote-sensing retrievals. Although studied sites are the best representation for each station and the surrounding area, mismatches between phenology products and ground-based observations at both temporal and spatial scales are unavoidable [56]. In addition, the monitoring method may lead to a mismatch with the ground observations; therefore, we used a flexible, repeatable, and realistic means (the piecewise logistic models) to monitor seasonal and inter-annual dynamics in vegetation from remote-sensing data at large scales. However, the method may also lead to an offset in some situations when the remote-sensing time series do not match the piecewise logistic models.

Besides, even though based on the same algorithm and mathematical curvature changes for the same spatial/vegetation type, phenological metrics derived from EVI and LAI time series may have different biophysical meanings. It seems that the SOS and EOS that are extracted from LAI are close to bud burst and leaf defoliation respectively, while the SOS and EOS that are extracted from EVI is close to leaf unfolding and leaf coloring respectively at deciduous broadleaved forest site. Thus, the SOS that is extracted from LAI is earlier than that from EVI, while the EOS that is extracted from LAI is later than that from EVI overall according to the analysis results.

In conclusion, both the LAI and the EVI have advantages in different vegetation types. It should be considered that uniting multisource data is an effective way to improve the accuracy and validity of remote-sensing phenology products. However, for evergreen forests and sparse vegetation, extracting phenological metrics is still challenging using the LAI or EVI time series based on piecewise logistic models. New methods or data sources must be adopted for these land cover types. In addition, due to the coarse spatial resolution of current remote-sensing phenology products, other plants are inevitably present within one square kilometer, which may lead to variability in phenological developmental stages and a weak relationship between remote-sensing data and ground observations. These findings are of great value for improving the spatial resolution of remote-sensing phenology products to promote their application and development in the future.

## Figures and Tables

**Table 1:** Information for ground phenological observation sites in China.

Station Name	Code	Vegetation Type	Dominant Species	Lon	Lat	Source	Years
Shenyang	SY	crop	rice	123.360	41.520	CERN	2004–2009
Jiutai	JT	crop	rice	125.800	44.170	CMA	2003–2010
Naiman	NM	grass	horsetail	116.676	43.550	CERN	2005–2010
Shapotou	SPT	shrub	herbage	105.003	37.470	CERN	2002–2012
Heshan	HS	ENF	Masson’s pine, cedar	112.900	22.681	CERN	2004–2009
Dinghushan	DHS	EBF	Castanea henryi, Schima superba, Aporosa yunnanensis, Cryptocarya chinensis, Acmena acuminatissima	112.539	42.144	CERN	2004–2009
Beijing	BJF	DNF	Chinese pine, larch	115.425	39.958	CERN	2003–2011
Changbaishan	CBS	DBF	Meng gu oak	128.109	41.403	CERN	2003–2010

**Table 2:** One-to-one correspondence between remote-sensing phenological metrics and phenophases observed from ground observations.

Vegetation Type	SOS	PS	PE	EOS
evergreen tree	bud burst	-	-	leaf coloring
deciduous tree	bud burst	-	-	leaf defoliation
shrub	bud burst	-	-	leaf defoliation
herb	emergence	-	-	withering
rice	regreening	heading	grain-filling	harvest
